# Can yield, soil C and aggregation be improved under long‐term conservation agriculture in the eastern Indo‐Gangetic plain of India?

**DOI:** 10.1111/ejss.13092

**Published:** 2021-02-18

**Authors:** Surajit Mondal, Janki Sharan Mishra, Shish Pal Poonia, Rakesh Kumar, Rachana Dubey, Santosh Kumar, Mausam Verma, Karnena K. Rao, Akram Ahmed, Sharad Dwivedi, Bhagwati Prasad Bhatt, Ram Kanwar Malik, Virender Kumar, Andrew McDonald

**Affiliations:** ^1^ ICAR – Research Complex for Eastern Region Patna India; ^2^ International Maize and Wheat Improvement Center (CIMMYT) Patna India; ^3^ International Rice Research Institute (IRRI) Los Baños Philippines; ^4^ Cornell University in the School of Integrative Plant Science Ithaca New York USA

**Keywords:** aggregate‐associated organic C, macropore, mean weight diameter, no‐tillage, soil organic carbon stock

## Abstract

Deteriorating soil health, diminishing soil organic carbon (SOC), development of subsurface hard compact layer and declining system productivity are barriers to achieving sustainable production in the traditional rice–wheat cropping system (TA) in the eastern Indo‐Gangetic Plain of India. Conservation agriculture (CA), which favours minimum soil disturbance, crop residue retention and crop diversification could be a viable alternative to the TA to address most of those major problems. With that in mind, a long‐term experiment is being implemented at ICAR‐RCER, Patna, Bihar, India, with four treatments: (a) TA, (b) full CA (fCA) and (c and d) partial CA (pCA1 and pCA2), differing in crop establishment methods, cropping system and crop residue management in a randomized complete block design. Measurement of soil health parameters was carried out in the 11th year of the experiment. The results revealed a beneficial effect of CA and 46 and 40% increase in SOC concentration and stock, respectively, under fCA over TA in the 0–7.5‐cm soil layer. The effect of partial CA (pCA1 and pCA2) was variable, but an increasing trend was always observed under pCA compared to TA. There was an enrichment in SOC content of aggregates under CA irrespective of size class; however, no relation was found between SOC content and aggregate diameter. The contribution of macroaggregates to SOC stock was larger (36–66%) under CA in the 0–7.5‐cm soil layer. Adoption of CA improved the macroaggregate content, MWD and GMD of aggregates, and aggregation ratio. Soil macropore content was greater under fCA, whereas other parameters were similar among treatments. The impact of CA was mostly limited to 0–7.5 cm soil layer and a maximum up to 15 cm soil depth while evaluation until 60 cm soil depth was realized. The yield of rice in CA was comparable to or higher than in TA, whereas the system rice equivalent yield was always higher (38–53%) under CA than under the conventional practices. Therefore, a CA‐based cropping system must be encouraged, to increase SOC status, improve aggregation stability and, consequently, sustain or increase system productivity, in order to achieve food and nutritional security in the eastern Indo‐Gangetic Plain of India.

**Highlights:**

- Effects of long‐term conservation agriculture (CA) on soil C, aggregation and yield were evaluated.
- CA improved SOC concentration and stock by 46 and 40%, as well as macroaggregate SOC stock by 36–66%.
- Macro‐aggregation and mean weight diameter improved in CA but was mostly limited to a shallow soil depth.
- CA can be promoted for sustainability of a rice–wheat system due to higher productivity (38–53%).

## 1 INTRODUCTION

Globally, agricultural soils are more potent for sequestering atmospheric carbon (C), and this could be one of the viable options for slowing down the pace of climate change. Moreover, an increased soil C pool not only augments productivity but also offers yield sustainability (Lal, 2004; Pan, Smith, & Pan, 2009). Sequestration of C in cropland is also crucial for achieving food security through sustainable development goals. Therefore, several resource conservation practices are recommended to achieve the environmental targets of less C emission, better soil health and better productivity in a sustainable manner (Paustian et al., 2016). Conservation agriculture, which promotes minimum soil disturbance, protects the soil by surface residue or cover crops and favours crop rotation, is one such management practice (Lal, 2004; Luo, Wang, & Sun, 2010). Adoption of minimum tillage or no‐tillage against conventional tillage has been considered as a successful approach for larger C stocks in soil (Paustian, Six, Elliott, & Hunt, 2000; Six et al., 2004).

Although no‐tillage (NT) has been suggested extensively for the sustainability of soil health and a better environment, its impact on soil organic C (SOC) is diverse, both temporally and spatially. Many researchers have reported a positive impact of NT on SOC (Francaviglia, di Bene, Farina, & Salvati, 2017; Veloso et al., 2018; Virto, Barré, Burlot, & Chenu, 2012), while at the same time many others have reported no effect (Corsi, Friedrich, Kassam, Pisante, & Sà, 2012; de Sant‐Anna et al., 2017; Dimassi et al., 2014). Most of the authors have argued that the adoption of NT with residue retention increases the SOC content in the upper soil layer up to 10 cm (Angers & Eriksen‐Hamel, 2008; Luo et al., 2010). Inversion of soil through CT allows the incorporation of surface residue within the soil profile, which otherwise gets accumulated near the soil surface in NT. This leads to smaller SOC concentrations in the deeper soil layer (>10 cm) under NT (Luo et al., 2010; Mondal, Chakraborty, Bandyopadhyay, Aggarwal, & Rana, 2020a). However, a significant gain in SOC stock under NT due to greater C concentration in the upper soil layer is not nullified by the marginal decrease in the subsequent lower layers. Even a single tillage operation in a long‐term no‐till field can undo the SOC accumulation over the previous years (Conant, Easter, Paustian, Swan, & Williams, 2007; Powlson et al., 2014; VandenBygaart, 2016). These wide spatiotemporal variations in the effect of NT on SOC stock could be due to complicated interactions among diverse climates, soil texture, cropping system, duration of the experiment, etc. (Luo et al., 2010).

A balance between inputs and outputs of organic C is important for SOC stocks in soil (Six et al., 2004). Virto et al. (2012) concluded that about 30% of the variability in SOC stock between NT and CT was due to differential C inputs. Some studies conducted at a regional scale also came to a similar conclusion (Franzluebbers, 2005; Liebig et al., 2005). More research on the cropping parameters that might favour SOC accumulation when implementing NT is also needed if the promotion of NT is considered while taking into account SOC sequestration. Inclusion of legumes in cropping systems can improve the SOC level in soils (Samal et al., 2017; Veloso et al., 2018), particularly with conservation agriculture (Lal et al., 2004). In contrast, Hernanz, López, Navarrete, and Sanchez‐Giron (2002) did not find any effect of legumes on SOC stock following the conversion of CT to NT.

The accumulation and turnover rate of SOC in response to different agricultural practices are largely connected to soil aggregates (Galantini, Senesi, Brunetti, & Rosell, 2004). Soil aggregates are vital for protection and sequestration of SOC and nearly 90% of the SOC build‐up occurs in soil aggregates (Sarker et al., 2018; Somasundaram, Reeves, Wang, Heenan, & Dalal, 2017). Different aggregate size classes offer a varying degree of physical protection against microbial decomposition to the associated SOC and are affected by different management practices such as tillage and crop residue retention (Xie et al., 2017). Organic matter facilitates the binding of soil particles and favours aggregate formation, and conversely aggregates physically protect the SOC by encapsulation. Macroaggregates, considered to be a predictor of tillage‐induced changes, play a dominant role in physically protecting the SOC and maintaining better soil health.

Soil aggregation, the spatial arrangement of soil particles and voids, is an important physical property and is imperative for soil fertility as it controls erosion and arbitrates soil aeration, water movement and retention (Six et al., 2004; Zhao, Chen, Hu, & Li, 2017). Thus, it has great bearing on root development, plant growth and crop productivity (Berisso et al., 2013). Aggregates are formed by various binding agents (e.g., organic substances, oxides of iron and aluminium, carbonates, etc.) and soil constituents simultaneously at multiple levels (Bronick & Lal, 2005; Six et al., 2004). Soil management, such as tillage and crop residue or straw management and seasonal variability, has the most direct bearing on aggregates, by either physical force or modifying the aggregation process (Huang et al., 2018; Spaccini, Piccolo, Mbagwu, Zena Teshale, & Igwe, 2002). Conventional tillage impairs the aggregation process directly by physically breaking down the aggregates (Six, Elliott, & Paustian, 2000; Somasundaram et al., 2017) and indirectly by altering the biochemical environment of the soil (Barto, Alt, Oelmann, Wilcke, & Rillig, 2010). Moreover, the fungal mycelium network is destroyed by repeated tillage operations (Borie et al., 2006). In contrast, no‐tillage promotes the formation of aggregates by omitting physical disturbance and favours the formation of continuous pores, especially biopores, by decaying crop residue or faunal activities (such as earthworms), which can affect the transport functions of soil (Hartmann, Zink, Fleige, & Horn, 2012).

The rice–wheat cropping system is practised in an area of about 13.5 M ha on the Indo‐Gangetic Plain, which is fundamentally important for the food security of the region (Jat et al., 2019). The puddling carried out during rice cultivation destroys the soil structure and is also reported to form a hard‐compact layer (Aggarwal, Choudhary, Singh, & Chakraborty, 2006; Mondal et al., 2019) that restricts root movement and impairs soil fertility. This cropping system is currently experiencing yield plateauing and therefore the sustainability of the system is at stake. Progressive soil degradation, residue burning, lower application of organic manures and imbalanced use of fertilizer are also posing serious problems for achieving food security. Therefore, the resource‐intensive conventional rice–wheat system needs to be modified with efficient management practices that are in harmony with soil quality, resource conservation, sustainability and profitability of the system. Hence, CA could be a better alternative and could address the problem of residue burning, soil health degradation, environmental pollution, labour scarcity and yield stagnation. Recently, the Government of India has targeted the eastern part of the country for the achievment of food security for the nation by ushering in the second Green Revolution (Mishra, Bhatt, Arunachalam, & Jat, 2020).

Although the information on the short‐ and medium‐term (<10 years) effects of CA on soil aggregation and SOC in the eastern Indo‐Gangetic Plain is available, research information is lacking on the long‐term (i.e., ≥10 years) impacts of CA on soil aggregate size distribution (dry sieving) and associated C in the subsoil layers (i.e., ≥20 cm). Moreover, most of the previous studies focused on soil properties and much less importance was given to system productivity and profitability. We hypothesized that the conversion of the traditional rice–wheat cropping system to diversified conservation agriculture improves soil physical health, amasses more SOC and increases the productivity and profitability of the system. Thus, the objectives were to assess the aggregate size distribution and associated OC, quantify SOC accumulation and evaluate the productivity under different tillage, residue management and cropping systems.

## 2 MATERIALS AND METHODS

### 2.1 Experimental site

A field experiment was initiated during November 2009 with a long‐term perspective, taking four treatments varying in the cropping system, tillage, establishment methods, residue and other managements in a completely randomized block design with three replications at the research farm of the Indian Council of Agricultural Research (ICAR) – Research Complex for Eastern Region (RCER), Patna, Bihar, India (25.58° N, 85.06° E) (Supplementary Figure S1). The climate of the region is subtropical monsoon, with an annual average rainfall of 1,130 mm. About 85% of the total rainfall occurs during the rainy season (June–September). The hottest and coldest months are June and January, with a mean temperature of 31.2 and 15.6°C, respectively. The soil was silty clay in texture and neutral to mild alkaline in reaction. The total organic carbon content was 8 g kg^−1^ at the start of the experiment (Laik et al., 2014).

**FIGURE 1:**
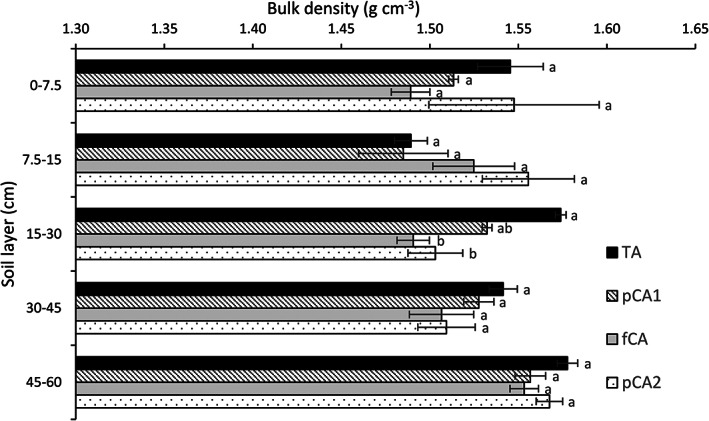
Effect of conservation agriculture on soil bulk density (g cm^−3^) in different soil layers. Vertical bars represent standard error of mean; bars with at least one common small letter are not statistically significant using Tukey's honest significant difference (HSD) at *p* < 0.05. fCA, full conservation agriculture; pCA1, partial conservation agriculture 1; pCA2, partial conservation agriculture 2; TA, traditional agriculture

### 2.2 Field experiment and treatments

The experiment was comprised of four treatments: (a) conventionally tilled wheat (*Triticum aestivum*) (CTW) – fallow – puddled transplanted rice (*Oryza sativa*) (PTR) (i.e., traditional agriculture [TA]), (b) NT wheat – NT greengram (*Vigna radiata*) – unpuddled machine transplanted rice (pCA1), (c) NT wheat – NT greengram – NT direct seeded rice (fCA) and (d) NT mustard (*Brassica juncea*) – NT maize (*Zea mays*) – NT direct seeded rice (pCA2). The details of the treatments are given in Table 1. Before the start of the experiment in 2009, laser levelling was carried out and puddled transplanted rice was grown to bring uniformity of the experimental field. After harvesting of rice, the field was divided into 12 plots of 2,000 m^2^ each.

**TABLE 1:** Details of tillage, seeding/planting methods, crop rotation and residue management under different treatments

Treatment name		Traditional agriculture	Partial conservation agriculture 1	Full conservation agriculture	Partial conservation agriculture 2
Treatment abbreviation		TA	pCA1	fCA	pCA2
Drivers of change		Business as usual (farmers’ practice)	To increase production and income through best management practices	To deal with the rising scarcity of water, energy and labour, degrading soil health (CA practice)	Futuristic, intensified and diversified for food and nutritional security and farm profitability
Tillage method	2009–2014	Wheat – Conventional tillage (CT) Rice –CT followed by puddling	Wheat – No–tillage (NT) Greengram – NT Rice – CT (puddled)	Wheat – NT Cowpea – NT Rice – NT	Potato/maize – CT Cowpea – NT Rice – CT (unpuddled)
	2014–2019		Wheat – NT Greengram – NT Rice – CT followed by machine transplanting (unpuddled)	Wheat – NT Greengram – NT Rice – NT	Mustard – NT Maize – NT Rice – NT
Seeding/planting method	2009–2014	Wheat – Drill seeding Rice – Transplanting	Wheat – Drill seeding Greengram – Drill seeding Rice – Transplanting	Wheat – Drill seeding Cowpea – Drill seeding Rice – Drill seeding (DSR)	Potato/maize – Dibbling Cowpea – Drill seeding Rice – Transplanting
	2014–2019		Wheat – Drill seeding Greengram – Drill seeding Rice – Transplanting	Wheat – Drill seeding Greengram – Drill seeding Rice – Drill seeding (DSR)	Mustard – Drill seeding Maize – Drill seeding Rice – Drill seeding (DSR)
Crop rotation	2009–2014	Wheat – Fallow – Rice	Wheat – Greengram – Rice	Wheat – Cowpea – Rice	Potato+maize – Cowpea – Rice
	2014–2019		Wheat – Greengram – Rice	Wheat – Greengram – Rice	Mustard – Maize – Rice
Crop residue management		Rice/wheat – Removed from ground level	Wheat – Removed Rice – Removed Greengram – Retained full and incorporated	Wheat – One third retained Rice – One third retained Cowpea – Retained full	Potato – Full, incorporated Maize – One third retained Cowpea – Full, incorporated Rice – One third incorporated
	2014–2019			Wheat – One third retained Rice – One third retained Greengram – Retained full	Mustard – One third retained Maize – One third retained Rice – One third retained

### 2.3 Soil sampling and analysis

Soil samples were collected in 2019 after harvesting of the rice crop (i.e., after completion of 10 years of the experiment). Soils were collected from eight randomly selected points in each plot using a soil auger, from 0–7.5, 7.5–15, 15–30, 30–45 and 45–60‐cm soil depths. Soils collected from each depth were aggregated to get a sample for each plot, which was used for aggregate analysis and SOC determination. Similarly, two soil cores were collected from each plot. One core was used for bulk density determination, whereas the other one was used in a hanging water column for pore‐size determination. The bulk samples were air‐dried, ground, passed through a 2‐mm sieve and stored for further physico‐chemical analysis.

#### 2.3.1 Bulk density

For determining the bulk density, core samples having a diameter and height of 5.3 and 5 cm, respectively, were collected with a core sampler. The soil cores were then dried at 100°C in a hot air oven until constant weight. The dry weight of the sample was divided by the volume of the core to get the soil bulk density.

#### 2.3.2 Size distribution of aggregates

Undisturbed composite samples were collected from the field in triplicate and used for aggregate analysis. After air‐drying, the samples were passed through a 4‐mm sieve and retained over a 2‐mm sieve to remove larger and smaller aggregates, respectively. A Yoder apparatus was used for wet sieving of aggregates (Yoder, 1936). A nest of five sieves having diameters of 2, 0.5, 0.25, 0.12 and 0.053 mm were used for the purpose. Briefly, 100 g air‐dried samples of aggregates were shaken over a 2‐mm sieve and capillary wetted for 10 min to minimize the slaking. Thereafter, the shaking operation was performed for 5 min with 35 cycles per minute. The soils collected in each sieve were transferred to a set of preweighed filter papers, oven‐dried at 65°C till constant weight and stored for SOC analysis. Sand correction for each aggregate class was performed. Various aggregation indices were computed as follows:

**(a) Macro‐, micro‐ and water‐stable aggregates:** In general, 0.25 mm is taken as the boundary between macro‐ and microaggregates. Aggregates retained over 2, 0.5 and 0.25 mm were summed to get the macroaggregates, whereas aggregates of 0.12 and 0.053 mm gave microaggregates. Water‐stable aggregates were obtained by adding macro‐ and microaggregates.

**(b) Mean weight diameter and geometric mean diameter:** MWD and GMD were calculated by the following formulae:

(1) $$\mathrm{MWD}\left(\mathrm{mm}\right)=\frac{\sum_{i=1}^{n}\left(X_{i}\times W_{i}\right)}{\sum_{i=1}^{n}W_{i}}.$$

(2) $$\mathrm{GMD}\left(\mathrm{mm}\right)=\exp\left[\frac{\sum_{i=1}^{n}\left(W_{i}\times \log X_{i}\right)}{\sum_{i=1}^{n}W_{i}}\right].$$

where Wi is the aggregates retained over ith sieve (in g) and Xi is the mean diameter of the size class (in mm).

**(c) Aggregate ratio (AR):** The aggregate ratio of soil was computed as:

(3) $$\mathrm{AR}=\frac{\text{Percent of water stable macro}-\text{aggregate}}{\text{Percent of water stable micro}-\text{aggregate}}.$$

**(d) Fractal dimension (D):** The fractal dimension of particle size distribution, which is used as an index of soil erodibility, was calculated by the following formula (Tyler & Wheatcraft, 1992):

(4) $$M\left(r<\mathrm{Ri}\right)/M_{T}={\left(\mathrm{Ri}/\text{Rmax}\right)}^{3-D}\;\text{or},D=3–\{\mathrm{Ln}\left(M\left(r<\mathrm{Ri}\right)/M_{T}\right)/\mathrm{Ln}\left(\mathrm{Ri}/\text{Rmax}\right).$$

According to Equation 1, 3‐D is the slope of the regression line Ln(M(r < Ri)/M_T_) as the Y‐axis and Ln(Ri/Rmax) as the X‐axis, and D is then calculated. Where M(r < Ri) is the cumulative percentage of particles of ith size r less than Ri, M_T_ is the total percentage, Ri is the particle radius (mm) of the ith size class and Rmax is the radius of the largest size class.

#### 2.3.3 Pore size distribution, field capacity, permanent wilting point and available water capacity

Undisturbed soil cores collected for bulk density were used for pore size distribution. Core samples were saturated by capillary intake of water and moisture content was determined to get the total porosity (TotP). The saturated soil cores were then placed over a hanging water column and a suction equivalent to a 60‐cm water column was applied. The volume of water extracted at this suction was equivalent to drained pore and was taken as macropore (MacP). Then the MacP was subtracted from total porosity to get microporosity (MicP). For determining the field capacity (FC) and permanent wilting point (PWP) of the water content, saturated samples placed over a porous plate were kept in a pressure chamber and 33 and 1,500 kPa pressure were applied for determination of the FC and PWP of the water content, respectively. After cessation of water movement, samples were removed from the pressure chamber, weighed and dried at 100°C for moisture content determination. The gravimetric moisture content was then multiplied by respective soil bulk density to get volumetric water content. The available water capacity (AWC) was calculated by subtracting the PWP water content from FC.

#### 2.3.4 Concentration and stock of SOC in bulk soil and aggregates

Soil organic carbon concentration of bulk soil, as well as aggregates, was analysed by dichromate oxidation in the presence of sulphuric acid, followed by titration with ferrous ammonium sulphate using a diphenylamine indicator (Walkley & Black, 1934). The SOC stock of bulk soil was calculated both by volume (depth) and mass basis. Similarly, aggregate SOC stock was also calculated. The following formulae were used for the calculations:

(5) $$\mathrm{SOC}\;\text{stock}\;\left(\mathrm{Mg}\;{\mathrm{ha}}^{-1},\mathrm{vol}.\text{basis}\right)=\mathrm{SOC}\;\left(g\;{\mathrm{kg}}^{-1}\right)\times \mathrm{BD}\;\left(g\;{\mathrm{cm}}^{-3}\right)\times \text{Depth}\;\left(\mathrm{cm}\right)\times 10^{-1}.$$

(6) $$\mathrm{SOC}\;\text{stock}\;\left(\mathrm{Mg}\;{\mathrm{ha}}^{-1},\text{mass}\text{s}\text{basis}\right)=\mathrm{SOC}\;\left(g\;{\mathrm{kg}}^{-1}\right)\times \mathrm{ESM}\;\left(\mathrm{kg}\;{\mathrm{ha}}^{-1}\right)\times 10^{-6}.$$

(7) $$\text{Aggregate}\;\mathrm{SOC}\;\text{stock}\;\left(\mathrm{Mg}\;{\mathrm{ha}}^{-1}\right)={\mathrm{SOC}}_{\mathrm{Agg}}\left(g\;{\mathrm{kg}}^{-1}\right)\times \mathrm{BD}\;\left(g\;{\mathrm{cm}}^{-3}\right)\times \text{Depth}\;\left(\mathrm{cm}\right)\times 10^{-6}\times P_{\mathrm{Agg}}).$$

where *BD* is mean bulk density of a particular depth, *SOC* is soil organic carbon concentration, *SOC_Agg_* is SOC concentration of aggregates, *P_Agg_* is the proportion of aggregates in total aggregates. The SOC measurement by the most accurate dry combustion method could not be performed due to unavailability of the facility and, therefore, the measured SOC could be underestimated.

### 2.4 Grain yield, system rice equivalent yield and profitability

Crops were harvested at maturity and grain yields at appropriate moisture contents were reported. For calculation of the system rice equivalent yield (SREY), the following equation was used. Among the treatments, number of crops taken per year varied and therefore to observe the effect of tillage and residue management, *SREY* for two crops (in rain and winter) were also calculated:

(8) $$\text{SREY}\;\left(\mathrm{Mg}\;{\mathrm{ha}}^{-1}\right)=\text{Rice yield}\;\left(\mathrm{Mg}\;{\mathrm{ha}}^{-1}\right)+\frac{\text{Yield of non rice crop}\;\left(\mathrm{Mg}\;{\mathrm{ha}}^{-1}\right)\times \mathrm{MSP}\;\text{of non rice crop}\;\left(\mathrm{INR}\;{\mathrm{Mg}}^{-1}\right)}{\mathrm{MSP}\;\text{of rice}\;\left(\mathrm{INR}\;{\mathrm{Mg}}^{-1}\right)}\ldots \ldots \ldots \ldots$$

where *MSP* is the minimum support price in Indian Rupees (*INR*), which is fixed by the Government of India from time to time.

For calculation of system profitability, all input costs for a particular crop year (winter‐summer‐rainy season) were summed to get the total cost of cultivation. Similarly, total income was obtained by multiplying grain/economic yield by the MSP of the respective crops. The cost of cultivation was then subtracted from total income to get net income.

### 2.5 Statistical analysis

Data were subjected to analysis of variance following a randomized block design by using the Statistical Analysis System (SAS, 2006) available at the Indian NARS Statistical Computing Portal (http://stat.iasri.res.in/sscnarsportal). Means were subjected to a significant difference at *p* < 0.05 by Tukey's honest significant difference (HSD) test. The MS Excel was used for basic calculation, interpretation and preparation of figures.

## 3 RESULTS

### 3.1 Bulk density

The effect of different treatments on soil BD was absent up to a 15‐cm soil depth (Figure 1). In the surface 0–7.5‐cm soil layer, the highest (1.55 g cm^−3^) and the lowest (1.49 g cm^−3^) BDs were noted in pCA2 and fCA, respectively. In the 15–30‐cm soil layer, TA recorded significantly higher BD (4.7–5.6%; *p* < 0.05) than fCA and pCA2. All other layers revealed no difference in BD among the different treatments.

### 3.2 Soil organic carbon and SOC stock

**FIGURE 2:**
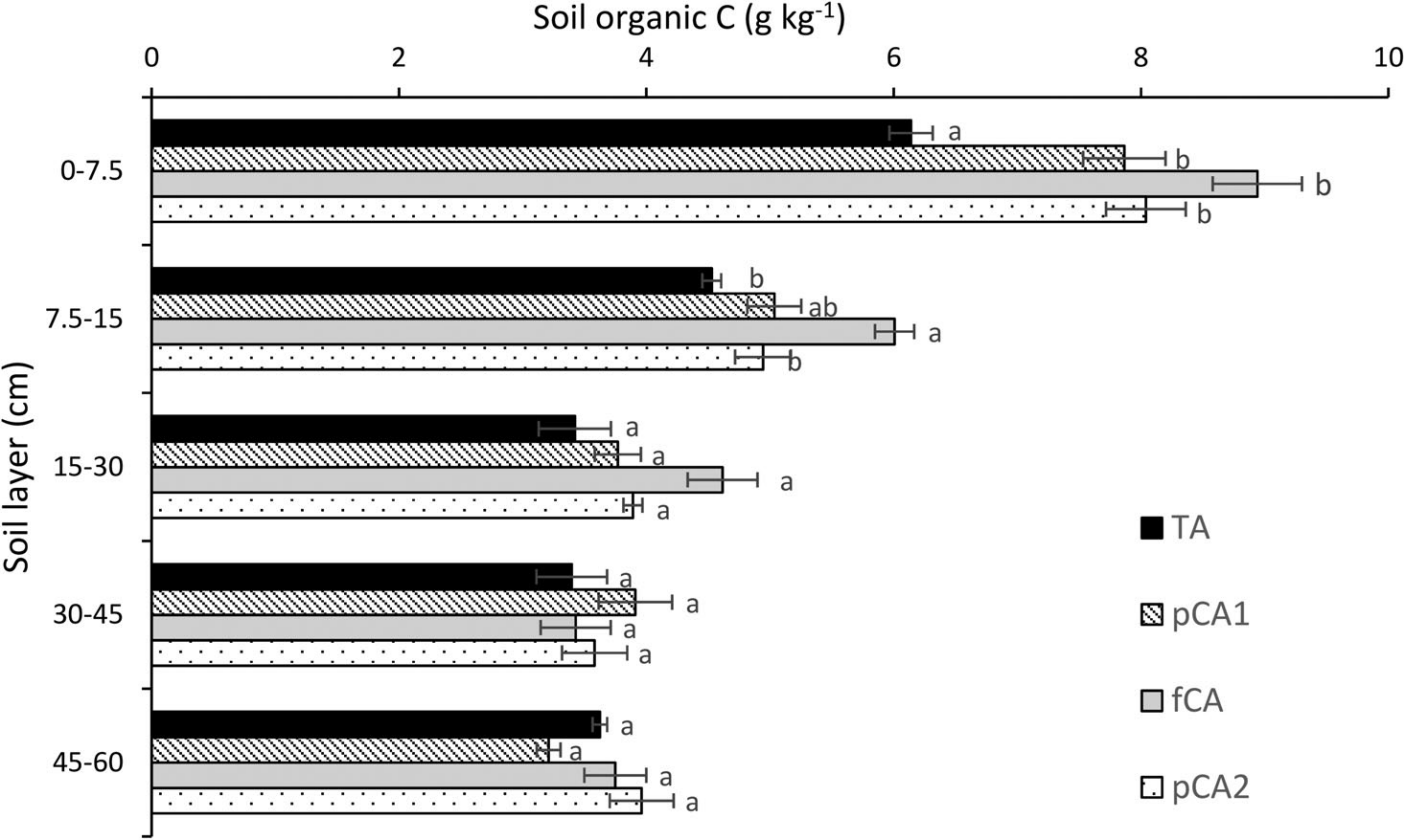
Soil organic C concentration (g kg^−1^) as affected by conservation agriculture. Vertical bars indicate standard error of mean; bars with at least one common small letter are not statistically significant using Tukey's honest significant difference (HSD) at *p* < 0.05. fCA, full conservation agriculture; pCA1, partial conservation agriculture 1; pCA2, partial conservation agriculture 2; TA, traditional agriculture

After 10 years of experimentation, the effect of different treatments was most prominent on SOC. In the 0–7.5‐cm soil layer, CA‐based treatment (fCA and pCA2) recorded 31–46% greater SOC than the conventional system (TA) (Figure 2). In the second layer (7.5–15 cm), fCA resulted in significantly larger SOC (22–33%; *p* < 0.05) than TA and pCA2 but was at a par with pCA1. However, no significant effect of treatment on SOC was noted beyond 15‐cm soil depth. SOC stock (both on a volume and mass basis) varied considerably due to different management practices (Table 2). SOC stock increased 40.3% due to full CA in comparison to TA in the surface layer. In the subsequent layer, fCA reported 18.9–35.6% larger SOC stock than the rest of the treatments. In the case of the mass basis of SOC stock, all CA practices (both partial and full) resulted in a 28.1–45.6% greater SOC stock than TA in the 0–7.5‐cm soil layer, whereas a 32.7% greater value was noted in fCA in the 7.5–15‐cm soil layer than for the conventional practices. After 15‐cm soil depth, SOC stock in both methods was similar for all the treatments. The total SOC stock for the 0–60‐cm soil profile was significantly larger, by 19.0 and 22.0%, in fCA than in TA on a volume and mass basis, respectively. However, none of the partial CA (pCA) treatments registered significantly greater SOC stock in comparison to TA.

**TABLE 2:** Soil organic C stock (Mg ha^−1^) in equivalent soil volume and mass basis under different levels of conservation agriculture in various soil layers after 10 years of adoption

Treatment	Equivalent soil volume basis					
	Soil layer (cm)					
	0–7.5	7.5–15	15–30	30–45	45–60	0–60
TA	7.12 b	5.06 b	8.08 a	7.86 a	8.58 a	36.70 b
pCA1	8.93 ab	5.60 b	8.66 a	8.97 a	7.49 a	39.66 ab
fCA	9.99 a	6.86 a	10.32 a	7.76 a	8.74 a	43.67 a
pCA2	9.36 ab	5.77 b	8.78 a	8.11 a	9.32 a	41.34 ab

	Equivalent soil mass basis					
	Soil mass (Mg ha^−1^)					
	~1,143	~1,136	~2,228	~2,282	~2,346	~9,194
TA	7.02 b	5.14 b	7.83 a	7.75 a	8.50 a	36.25 b
pCA1	8.99 a	5.72 ab	8.62 a	8.93 a	7.53 a	39.79 ab
fCA	10.22 a	6.82 a	10.56 a	7.82 a	8.79 a	44.22 a
pCA2	9.19 a	5.62 b	8.90 a	8.17 a	9.30 a	41.18 ab

*Note*: Means with at least one common small letter are not statistically significant using Tukey's honest significant difference (HSD) at *p* < 0.05.

Abbreviations: fCA, full conservation agriculture; pCA1, partial conservation agriculture 1; pCA2, partial conservation agriculture 2; TA, traditional agriculture.

### 3.3 Aggregate distribution and aggregation indices

In the 0–7.5‐cm soil layer, CA‐based treatments (both partial and full) resulted in 13.6–26.6% larger (*p* < 0.05) MacA content than TA (Table 3). However, the effect was absent in the subsequent soil layer. A reverse trend was noted for MicA, and TA recorded significantly larger MicA content in both the 0–7.5 and 7.5–15‐cm soil layers. In the case of WSA, fCA reported 8.2% more water stable aggregates than TA, but was at a par with other treatments in the surface layer. A higher aggregate ratio was observed under conservation agriculture than TA both in the 0–7.5‐cm (53.5–83.6%; *p* < 0.05) and 7.5–15‐cm (27.3–55.3%) soil layers. The largest and the smallest MWDs were registered for fCA and TA, respectively, and the order was fCA > pCA2 > pCA1 > TA in the surface soil layer. In the following layer, fCA resulted in a larger (31.6–71.2%; *p* < 0.05) MWD of aggregates than TA and pCA2. A similar trend was noted for GMD. The fractal dimension of particle size distribution, which is used as an index of soil erodibility, was similar among different treatments in the surface layer, but a slight variation was noted in the second layer and pCA2 resulted in a lower FD value than TA. None of the aggregate fractions or indices exhibited any difference beyond 15‐cm soil depth.

**TABLE 3:** Soil aggregation parameters and indices as affected by conservation agriculture in different soil layers after 10 years of adoption

Treatment	Aggregation parameter/index						
	MacA	MicA	WSA	AR	MWD	GMD	FD
	%				(mm)		
0–7.5‐cm soil layer							
TA	58.0 c	27.3 a	85.2 b	2.13 c	0.77 d	0.80 c	3.20 a
pCA1	67.2 ab	18.8 b	86.0 ab	3.59 ab	1.29 c	0.96 b	3.18 a
fCA	73.4 a	18.8 b	92.2 a	3.91 a	1.68 a	1.05 a	3.20 a
pCA2	65.9 b	20.2 b	86.1 ab	3.27 b	1.45 b	0.99 b	3.18 a
7.5–15‐cm soil layer							
TA	63.5 a	25.3 a	88.8 a	2.53 b	0.73 c	0.76 c	3.21 a
pCA1	67.2 a	21.0 ab	88.2 a	3.22 ab	1.11 ab	0.91 a	3.20 ab
fCA	71.2 a	18.3 b	89.5 a	3.93 a	1.25 a	0.94 a	3.20 ab
pCA2	69.5 a	20.3 ab	89.8 a	3.45 ab	0.95 b	0.85 b	3.19 b
15–30‐cm soil layer							
TA	62.0 a	25.5 a	87.4 a	2.44 a	0.95 a	0.86 a	3.21 a
pCA1	69.5 a	24.4 a	93.9 a	2.86 a	0.93 a	0.83 a	3.21 a
fCA	71.5 a	20.1 a	91.6 a	3.67 a	0.92 a	0.85 a	3.20 a
pCA2	62.7 a	25.0 a	87.7 a	2.53 a	0.84 a	0.81 a	3.18 a
30–45‐cm soil layer							
TA	65.2 a	20.3 a	85.5 a	3.26 a	0.79 a	0.83 a	3.19 a
pCA1	69.4 a	22.3 a	91.7 a	3.24 a	0.88 a	0.83 a	3.20 a
fCA	65.2 a	21.7 a	86.9 a	3.16 a	0.88 a	0.86 a	3.19 a
pCA2	63.5 a	20.5 a	84.0 a	3.13 a	0.82 a	0.84 a	3.19 a
45–60‐cm soil layer							
TA	61.3 a	23.2 a	84.5 a	2.71 a	0.80 a	0.83 a	3.19 a
pCA1	62.4 a	25.3 a	87.7 a	2.48 a	0.85 a	0.81 a	3.18 a
fCA	66.5 a	21.9 a	88.4 a	3.08 a	0.89 a	0.84 a	3.19 a
pCA2	66.2 a	23.6 a	89.7 a	2.86 a	0.81 a	0.81 a	3.20 a

*Note*: Means with at least one common small letter are not statistically significant using Tukey's honest significant difference (HSD) at *p* < 0.05.

Abbreviations: AR, aggregate ratio; fCA, full conservation agriculture; FD, fractal dimension; GMD, geometric mean diameter of aggregate; MacA, macroaggregate; MicA, microaggregate; MWD, mean weight diameter of aggregate; pCA1, partial conservation agriculture 1; pCA2, partial conservation agriculture 2; TA: traditional agriculture; WSA: water stable aggregate.

### 3.4 Aggregate‐associated organic carbon (ASOC)

**FIGURE 3:**
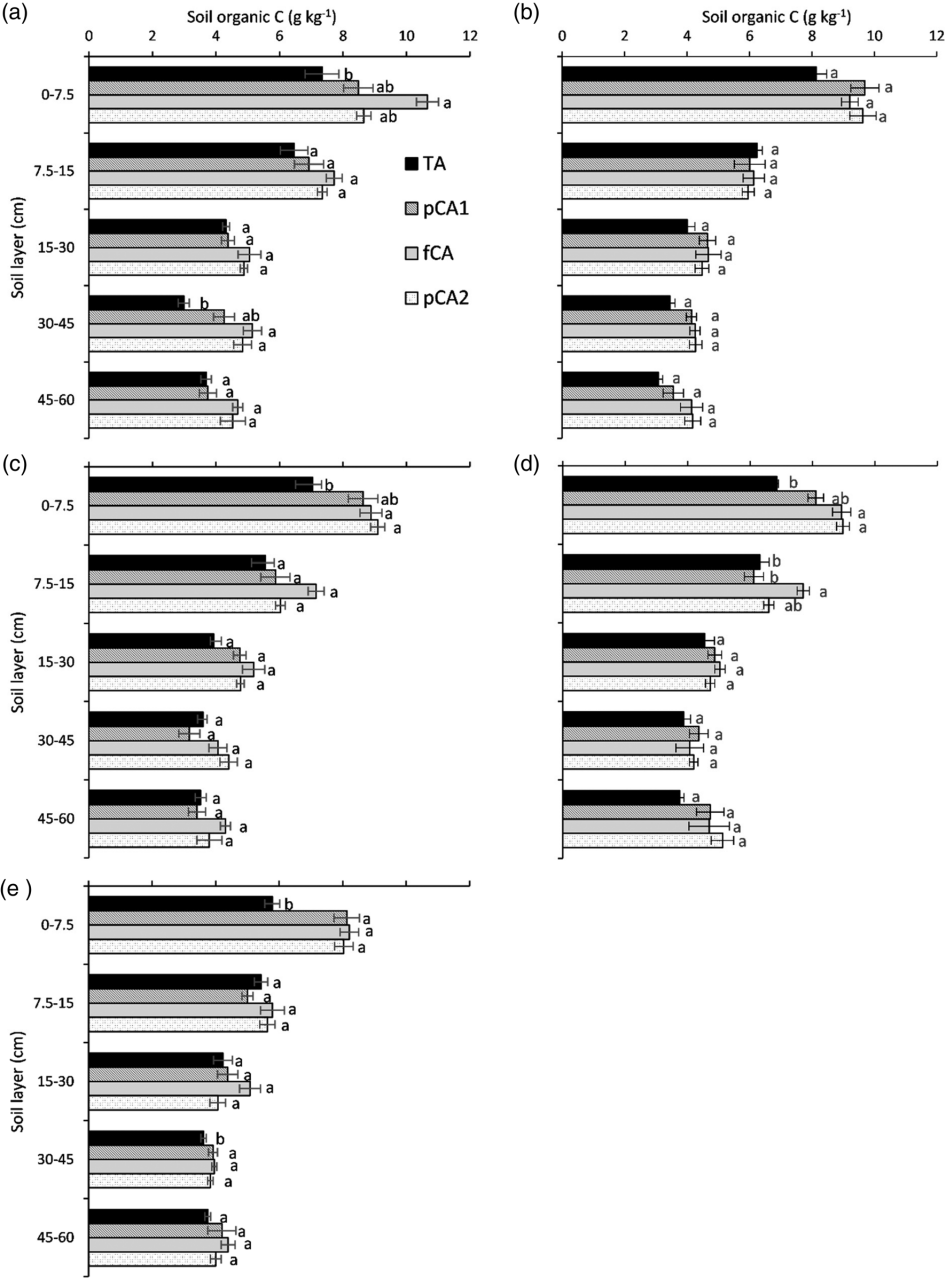
Effect of conservation agriculture on aggregate‐associated carbon (g kg^−1^) in different aggregate size classes: (a) 2–4, (b) 0.5–2, (c) 0.5–0.25, (d) 0.12–0.25 and (e) 0.053–0.12 mm. Vertical bars represent standard error of mean; bars with at least one common small letter are not statistically significant using Tukey's honest significant difference (HSD) at *p* < 0.05. fCA, full conservation agriculture; pCA1, partial conservation agriculture 1; pCA2, partial conservation agriculture 2; TA, traditional agriculture

**FIGURE 4:**
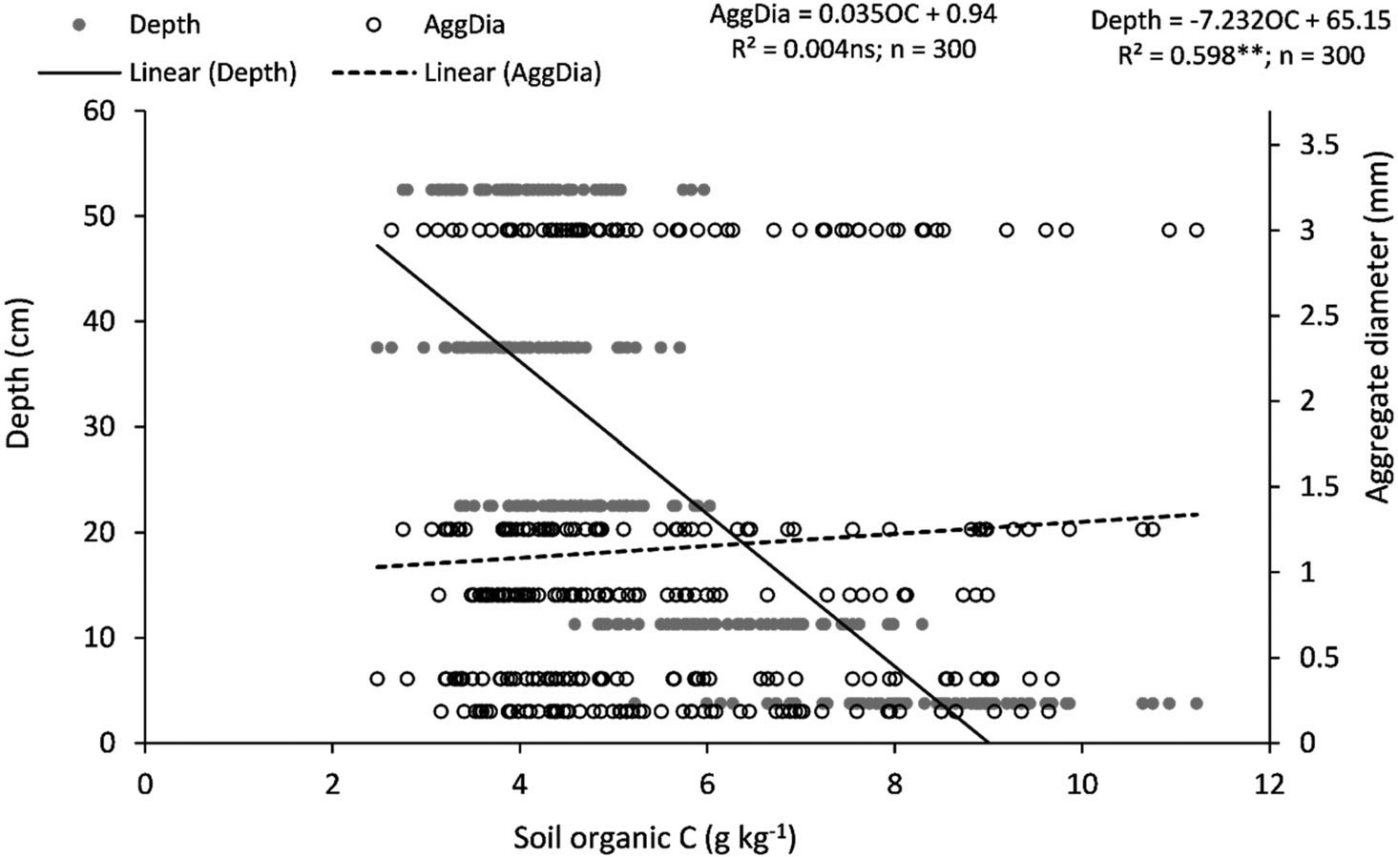
Relationship of soil organic carbon (SOC) concentration with aggregate diameter and soil depth. AggDia, aggregrate diameter

**FIGURE 5:**
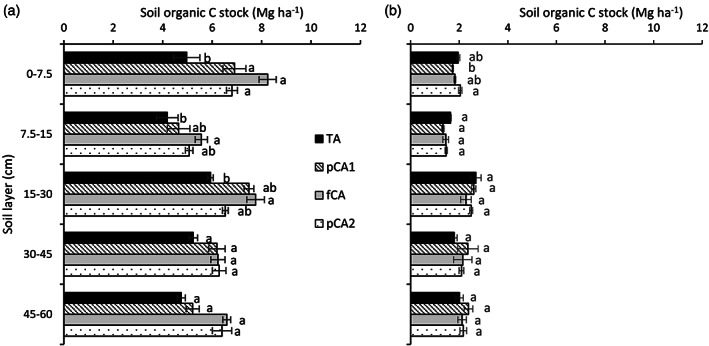
Aggregate‐associated organic C stock (Mg ha^−1^) in (a) macro‐ and (b) microaggregates. Vertical bars represent standard error of mean; bars with at least one common small letter are not statistically significant using Tukey's honest significant difference (HSD) at *p* < 0.05. fCA, full conservation agriculture; pCA1, partial conservation agriculture 1; pCA2, partial conservation agriculture 2; TA, traditional agriculture

For larger macroaggregates (>2 mm), the highest SOC content was noted in fCA and it was 45.2% larger than in TA in the 0–7.5‐cm soil layer (Figure 3). In the 30–45‐cm soil layer, TA registered significantly less (38.2–41.9%) ASOC than fCA and pCA2. Irrespective of depth, ASOC content in 0.5–2‐mm aggregates was similar in all treatments. For smaller macroaggregates (0.25–0.5 mm), fCA and pCA2 resulted in a larger (26.1–29.1%) ASOC content than TA. Except for the surface layer, none of the other soil layers registered any difference in ASOC content. For large microaggregates (0.1–0.25 mm), treatments that received full CA only had greater ASOC than conventional practices in the 0–7.5 and 7.5–15‐cm soil layers. In the case of small microaggregates, TA registered a 28.0–29.5 and 5.4–8.5% less ASOC than the rest of the treatments in the 0–7.5 and 30–45‐cm soil layers, respectively. The relationship of ASOC content with the aggregate diameter and soil depth was computed (Figure 4) and it has been observed that aggregate diameter had a non‐significant effect (R^2^ = 0.004) on ASOC, whereas soil depth played a pivotal role in ASOC content (R^2^ = 0.598; *p* < 0.01). The aggregate C stock was calculated and prominent differences were observed among treatments (Figure 5). In the surface layer, all treatments that received either partial or full CA had significantly larger (36.8–65.8%; *p* < 0.05) macroaggregate SOC stock than TA. The trend was similar up to 30‐cm soil depth but the magnitude decreased and only fCA had a larger (30.7–32.9%) MacA SOC stock than the conventional treatment. The impact of different treatments on MicA SOC stock was absent except for the 0–7.5‐cm soil layer, where fCA2 resulted in a largerstock (17.9%; *p* < 0.05) than fCA1. Irrespective of treatments, the MAC SOC stock was two to four times greater than the MicA SOC stock.

### 3.5 Pore size distribution and water content

The largest amount of MacP was noted in fCA and it was greater by 26.7% (*p* < 0.05) than in TA in the 0–7.5‐cm soil layer (Table 4). In the next layer, the effect was absent and again in the 15–30‐cm soil layer fCA recorded a larger (12.2–17.4%) macropore number than the rest of the treatments. In the case of micropores and total pores, all treatments registered a similar value throughout the soil profile. The field capacity moisture content was larger in TA than in most of the treatments in 0–7.5‐cm soil layer and a reverse trend was noted in the next layer. Irrespective of soil depth, moisture content at the permanent wilting point and available water capacity were similar in all treatments.

**TABLE 4:** Soil porosity and soil water retention characteristics as affected by different levels of conservation agriculture in different soil layers after 10 years of adoption

Treatment	Soil porosity			Soil water retention		
	MacP	MicP	TotP	FC	PWP	AWC
	%			(%, v v^−1^)		
0–7.5‐cm soil layer						
TA	8.6 b	33.3 a	41.9 a	44.6 a	26.8 a	17.8 a
pCA1	9.0 ab	34.0 a	43.0 a	38.4 c	24.3 a	14.1 a
fCA	10.9 a	33.2 a	44.1 a	41.1 b	25.7 a	15.4 a
pCA2	8.9 ab	34.7 a	43.6 a	42.7 ab	25.7 a	17.0 a
7.5–15‐cm soil layer						
TA	9.1 a	32.9 a	42.0 a	38.3 b	22.9 a	15.4 a
pCA1	9.0 a	33.1 a	42.2 a	39.1 b	22.6 a	16.4 a
fCA	10.1 a	32.3 a	42.4 a	44.8 a	24.9 a	19.9 a
pCA2	8.6 a	33.5 a	43.2 a	44.8 a	25.7 a	19.1 a
15–30‐cm soil layer						
TA	8.1 b	33.6 a	41.6 a	43.6 a	24.5 a	19.1 a
pCA1	7.7 b	33.5 a	41.2 a	40.7 ab	24.3 a	16.3 a
fCA	11.0 a	33.2 a	44.2 a	39.5 b	24.2 a	15.3 a
pCA2	8.0 b	33.4 a	41.4 a	40.1 ab	23.5 a	16.6 a
30–45‐cm soil layer						
TA	9.1 a	32.3 a	41.4 a	44.0 a	23.6 a	20.4 a
pCA1	8.0 a	31.4 a	39.4 a	45.8 a	24.5 a	21.3 a
fCA	7.9 a	31.6 a	39.5 a	43.8 a	25.7 a	18.0 a
pCA2	7.3 a	34.7 a	42.0 a	42.8 a	25.4 a	17.4 a
45–60‐cm soil layer						
TA	9.2 a	34.1 a	43.3 a	45.9 a	23.7 a	22.2 a
pCA1	8.3 a	33.6 a	41.9 a	46.9 a	25.0 a	21.9 a
fCA	6.6 a	34.1 a	40.8 a	47.2 a	22.9 a	24.2 a
pCA2	7.0 a	34.1 a	41.1 a	46.6 a	22.8 a	23.8 a

*Note*: Means with at least one common small letter are not statistically significant using Tukey's honest significant difference (HSD) at *p* < 0.05.

Abbreviations: AWC, available water capacity; FC, field capacity; fCA, full conservation agriculture; MacP, macropore; MicP, micropore; pCA1, partial conservation agriculture 1; pCA2, partial conservation agriculture 2; PWP, permanent wilting point; TA, traditional agriculture; TotP, total pore.

### 3.6 Grain yield, system productivity and profitability

**FIGURE 6:**
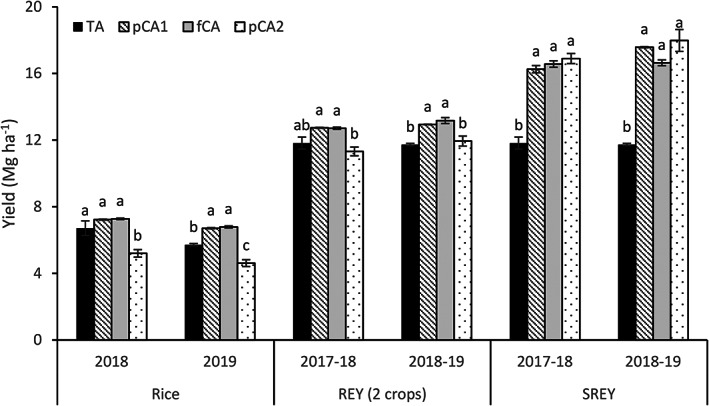
Yield of rice, rice equivalent yield of two crops and system rice equivalent yield in Mg ha^−1^. Vertical bars represent standard error of mean; bars with at least one common small letter are not statistically significant using Tukey's honest significant difference (HSD) at *p* < 0.05. fCA, full conservation agriculture; pCA1, partial conservation agriculture 1; pCA2, partial conservation agriculture 2; REY, rice equivalent yield; SREY; system rice equivalent yield; TA, traditional agriculture

The yield data of the last 2 years have been presented in Figure 6, and it can be observed that in both years fCA and pCA1 had a similar (2018) or higher (2019) rice yield compared to CA. The pCA2 had a consistently lower yield (22.6–28.6%; *p* < 0.05) than the rest of the treatments. To bring uniformity in crops grown in a year, rice equivalent yields (REY) for two crops (rainy and winter season crops) were calculated. The REY (two crops) was again similar or higher in fCA and pCA compared to TA; however, pCA2 resulted in a similar yield of TA but was significantly lower than the other two treatments. The SREY was always higher (37.5–53.3%; *p* < 0.05) in treatments where partial or full CA was adopted in comparison to TA. The total cost of cultivation (COC) varied between US$ 1584 (TA) and US$ 1963 (pCA1) among different treatments (Table 5). Both partial and full CA recorded a higher (8.3–24.0%) total COC than TA; however, for a single crop, CA always recorded lower (2–30%) COC than TA. The income from grain yield was highest in pCA2 (US$ 4,546), which was closely followed by pCA1 (US$ 4,411) and fCA (US$ 4,326), whereas the lowest income was achieved in TA (US$ 3,068). Similarly, net income was always higher (65–76%) in CA than TA.

**TABLE 5:** Crop‐wise grain yield, amount of crop residue retained, cost of cultivation, income from grain yield and net income as affected by different levels of conservation agriculture

Crop	Rice		Wheat		Greengram		Mustard		Maize		Total (avg. of 2 years)	
Year	2018	2019	2017–18	2018–19	2018	2019	2017–18	2018–19	2018	2019	In INR	In US$^b^
Grain yield (Mg ha^−1^)												
TA	6.72	5.72	5.15	5.93	—	—	—	—	—	—	—	—
pCA1	7.23	6.71	5.56	6.14	1.10	1.21	—	—	—	—	—	—
fCA	7.28	6.78	5.48	6.30	1.21	0.90	—	—	—	—	—	—
pCA2	5.20	4.61	—	—	—	—	2.68	3.17	6.85	6.45	—	—
Amount of crop residue retained (Mg ha^−1^)												
TA	0	0	0	0	—	—	—	—	—	—	0^a^	0^a^
pCA1	2.11	1.96	2.09	2.38	2.62	2.84	—	—	—	—	6.82	7.18
fCA	2.46	2.14	2.07	2.53	2.49	2.58	—	—	—	—	7.02	7.25
pCA2	1.73	1.67	—	—	—	—	1.27	1.57	3.78	3.61	6.78	6.85
Cost of cultivation (INR ha^−1^)												
TA	60,440	—	47,905	—	—	—	—	—	—	—	108,345	1,584
pCA1	59,240	—	37,761	—	37,319	—	—	—	—	—	134,320	1,963
fCA	42,275	—	37,761	—	37,319	—	—	—	—	—	117,355	1,715
pCA2	42,275	—	—	—	—	—	38,377	—	51,738	—	132,390	1,935
Income from grain yield (INR ha^−1^)												
TA	117,524	103,748	89,319	109,144	—	—	—	—	—	—	209,868	3,068
pCA1	126,449	121,787	96,466	113,049	61,546	84,172	—	—	—	—	301,735	4,411
fCA	127,313	123,138	95,117	115,900	67,476	62,937	—	—	—	—	295,941	4,326
pCA2	91,008	83,727	—	—	—	—	107,019	133,048	97,645	109,568	311,008	4,546
Net income (INR ha^−1^)												
TA	57,084	43,308	41,414	61,239	—	—	—	—	—	—	101,523	1,484
pCA1	67,209	62,547	58,705	75,288	24,227	46,853	—	—	—	—	167,415	2,447
fCA	85,038	80,863	57,356	78,139	30,157	25,618	—	—	—	—	178,586	2,610
pCA2	48,733	41,452	—	—	—	—	68,642	94,671	45,907	57,830	178,618	2,611

Abbreviations: fCA, full conservation agriculture; pCA1, partial conservation agriculture 1; pCA2, partial conservation agriculture 2; TA, traditional agriculture.

^a^ Crop residue retained in Mg ha^−1^ for 2017–18 and 2018–19.

^b^ 1 US$ = 68.4113 INR (average exchange rate for 2018).

## 4 DISCUSSION

### 4.1 Bulk density

The BD is the most common soil physical property and often used to appraise the effect of tillage and residue. No differences in soil BD were observed up to 15 cm soil depth. However, a greater BD was noted in TA than in fCA in the 15–30‐cm soil layer. This could be attributed to the puddling during rice cultivation in TA. Development of a subsurface soil layer has been extensively reported in the rice–wheat cropping system of the Indo‐Gangetic Plain (Aggarwal et al., 2006; Mondal et al., 2019). Recent global meta‐analyses also reported a contrasting impact of NT on soil BD; Li, Li, Cui, Jagadamma, and Zhang (2019) reported a 1.4% increase (*p* < 0.05) in BD under CA, whereas Mondal et al. (2020b) noted a similar BD in CT and NT.

### 4.2 Soil organic C

The impact of conversion of TA to CA on SOC was visible in the soil profile, particularly in the upper soil layers. Complete adoption of CA improved the SOC concentration by 46 and 33% in 0–7.5 and 7.5–15‐cm soil layers, respectively, whereas partial CA was most effective in the surface layer. Maximum differences in SOC concentration among treatments were noted in the surface layer and beyond the 7.5‐cm soil depth the magnitude of the difference reduced considerably. This wide variation in change in SOC concentration between the two layers could be due to retention of crop residue (~7 Mg ha^−1^) and suggests the stratification of SOC in the soil profile. Non‐disturbance of soil under CA allows the accumulation of organic matter in the surface soil layer (Franzluebbers & Steiner, 2016; Hernanz et al., 2002), which otherwise gets distributed within the plough layer under TA. Erosion control, water and nutrient conservation due to surface residue in NT further aggravate the stratification. Unlike SOC concentration, the SOC stock, which is a product of SOC concentration and soil BD, was positively impacted by full CA throughout the soil profile (0–60 cm). The largest gain (40.3% [volume basis] and 45.6% [mass basis]) in SOC stock was observed in the 0–7.5‐cm soil layer, which reduced gradually with increasing soil depth and became similar beyond 15 cm soil depth. The effect of partial CA on SOC stock was inconsistent and was similar to that in TA. These findings are in good agreement with those of others (Haddaway et al., 2017; Meurer, Haddaway, Bolinder, & Kätterer, 2018; Mondal, Chakraborty, et al., 2020a; Veloso et al., 2018); however, depths of impact varied. Haddaway et al. (2017) noted a significant treatment effect up to a depth of 30 cm, whereas the effect disappeared when deeper soil depths were considered (0–150 cm). In a long‐term experiment (43 years), Ussiri and Lal (2009) reported a threefold increase in SOC stock in the 0–15‐cm soil layer, whereas a 15% reduction was noted in the 15–30‐cm soil depth. Moreover, Meurer et al. (2018) observed an increase in SOC stock in the 0–45‐cm soil layer, whereas West and Post (2002) reported an effective depth of 7 cm for ~85% of the C sequestration. Recent meta‐analyses suggest a mere redistribution of soil C under NT, with a net gain in shallow depths and a net loss in deeper layers (Luo et al., 2010; Mondal, Chakraborty, et al., 2020a). Therefore, estimation of SOC stock for upper soil layers (say up to 30 cm) could cause overestimation of SOC accumulation under NT (Aguilera, Lassaletta, Gattinger, & Gimeno, 2013; Haddaway et al., 2017; Mondal, Chakraborty, et al., 2020a; Virto et al., 2012). However, we have not noticed any decrease in SOC content in the deeper soil layer.

Differences in SOC stock under NT or CT can be linked to variable yield (Virto et al., 2012) and, therefore, net primary productivity, which decides the amount of organic matter inputs to the soil in the form of crop residue (Kumar et al., 2021). Under NT, the crop residue remains on the surface and is expected to influence the SOC concentration in the upper soil layers only, while the same likely to get incorporated to greater soil depth under CT and thereby alter the SOC balance of greater soil depth than NT (Meurer et al., 2018). Several researchers have highlighted the beneficial effect of NT on net primary productivity (Olson & Ebelhar, 2009; So, Grabski, & Desborough, 2009), while at the same time opposite or null effects are also reported in the literature (Giller, Witter, Corbeels, & Tittonell, 2009; Wang, Cai, Hoogmoed, Oenema, & Perdok, 2006). Therefore, the SOC stock can be increased significantly where NT causes increased production (Follett, Castellanos, & Buenger, 2005). The SOC level of soil can be improved considerably by including a legume in the cropping systems (Samal et al., 2017; Veloso et al., 2018), particularly with conservation agriculture (Lal et al., 2004). In contrast, no role of legumes in SOC stock following the conversion of CT to NT was reported by Hernanz et al. (2002).

### 4.3 Soil aggregation

A notable difference in aggregation characteristics was observed among the treatments due to long‐term tillage and residue management practices. Adoption of CA, especially full CA, improved the macroaggregate content, MWD and GMD of aggregates, and the aggregation ratio, in comparison to conventional tillage in the surface soil layer. However, an identical amount of WSA aggregate was noted among the treatments, except for full CA. This could be attributed to the minimum soil disturbance and crop residue retention under CA. Absence of tillage excludes the possibility of physical disruption of soil aggregates due to tillage implements (Barto et al., 2010), and soil organic matter remains protected within the aggregates and is less prone to oxidation. Consequently, the enhanced SOC level favours aggregation and confers stability on the aggregates (Denef & Six, 2005). The TA could also negatively affect the earthworm population (Barto et al., 2010). The MWD of aggregates, which is widely accepted as a soil structural indicator (Kemper & Chepil, 1965), was improved under CA and this implies an improvement in soil stability, which is crucial for soil aeration, root elongation and water movement (Mondal, Chakraborty, et al., 2020a). Several studies have also reported a similar finding of higher aggregation under NT in comparison to CT (Mchunu, Lorentz, Jewitt, Manson, & Chaplot, 2011; Sheehy, Regina, Alakukku, & Six, 2015). Despite considerable changes in MacA and MicA, minimal variation in WSA was observed among treatments and this indicates a redistribution of macro‐ and microaggregates and a steady turnover rate of WSA. Our findings are in parallel with those of Hati et al. (2015) and Mondal, Poonia, et al. (2020b).

Differential organic matter content in tropical soils could be a deciding factor for variation of different aggregates and related indices (Castro Filho, Lourenço, Guimarães, & Fonseca, 2002). Crop residue retained on the soil surface has twofold effects on soil aggregation. Firstly, it acts as a barrier between soil aggregates and external forces such as raindrop impact (Blanco‐Canqui & Lal, 2009). Secondly, organic compounds such as polysaccharides, organic acids, glomalin, etc., released during microbial decomposition, act as binding agents during macroaggregate formation and offer stability to the newly formed aggregates (Choudhury et al., 2014; Somasundaram et al., 2017). Regular residue addition improves substrate availability and water retention, which in turn favour microbial activity (Balota & Filho, 2004; Denef & Six, 2005). The added crop residue acts as a hotspot of microbial activity and enhances earthworm activity, which is believed to have a beneficial effect on soil structure (Nyamadzawo, Nyamangara, Nyamugafata, & Muzulu, 2009). The contribution of soil biota to soil aggregation has also been documented by Lehmann, Zheng, and Rillig (2017) through a global meta‐analysis. The long‐term impact of tillage and residue management was mostly limited to the upper soil layers and no effects were noted on any of the soil aggregation parameters beyond 15 cm soil depth.

### 4.4 Aggregate‐associated organic C

The effect of differing tillage, residue and cropping systems on ASOC was mostly visible in the surface 0–7.5‐cm soil layer. The adoption of either full or partial CA increased the ASOC in comparison to TA; however, it was largely significant for fCA and pCA2. Crop residue retention or incorporation could be the probable reason for higher ASOC in the surface layer and NT further facilitates the accumulation (Jat et al., 2019; Six, Conant, Paul, & Paustian, 2002). Irrespective of aggregate size class, ASOC increased under CA in the surface soil layer. Our findings are in agreement with those of others (Choudhury et al., 2014; Jat et al., 2019); however, they contradict the outcomes reported by Pinheiro, Pereira, and Anjos (2004) and Madari, Machado, Torres, de Andrade, and Valencia (2005), who have reported an increase in ASOC for MacA only. SOC plays a dominant role during aggregate formation and gets encapsulated within the macroaggregates. The entrapped SOC is less accessible to the soil microbes and has a longer turnover time. Thus, CA, which favours less soil disturbance and residue retention, resulted in an accrual of SOC (Six et al., 2004). In contrast to CA, TA physically disrupts the aggregates and favours the microbial decomposition of SOC, resulting in lower ASOC (Luo et al., 2010; Pinheiro et al., 2004). A non‐significant regression coefficient between SOC and aggregate diameter indicates the independent ability of the two variables. However, SOC was greatly influenced by the depth and a steep slope was observed. Unlike concentration, the aggregate SOC stock varied considerably among the treatments up to 30‐cm soil depth. Macroaggregates contributed the most towards SOC stock and CA resulted in a higher MacA SOC stock than TA. The SOC carried by MicA was two to four times less than that carried by MacA and was almost similar in all treatments irrespective of soil depth. Increased SOC content promoted macro‐aggregation, which in turn retained more SOC under CA. Similar findings have also been reported by others (Kan et al., 2020; Wang et al., 2019). In contrast, John, Yamashita, Ludwig, and Flessa (2005) and Du, Ren, Hu, Zhang, and Blanco‐Canqui (2013) reported MicA as the main carrier of SOC.

### 4.5 Soil porosity and water content

Soil porosity, which controls soil aeration, is affected by the structural stability of soil in spatial as well as in temporal domains as a result of various management practices. An efficient and stable pore network is expected to form under long‐term management practices (Horn, 2004). The complete adoption of CA over TA improved macro‐porosity by 26.7 and 35.8% in the 0–7.5 and 15–30‐cm soil layers. This could be attributed to greater earthworm activity and decayed root channels under fCA. Absence of tillage and residue retention over a longer period (10 years) improved the physical structure of soil, resulting in a larger MacP under fCA, whereas puddling under TA destroys the aggregates and sometimes facilitates the formation of a subsurface hard layer, which is therefore believed to have a lower number of macropores. Similar findings have also been reported by many other authors (He et al., 2011; Mondal et al., 2013). Partial adoption of CA did not improve macroporosity. On the contrary, larger macropore and total pore levels were noted under CA in our earlier investigation (after 5 years) (Mondal, Poonia, et al., 2020b). Therefore, the period of adoption could be an important factor in determining pore size distribution. Microporosity and total porosity were similar in all treatments and this could be ascribed to the presence of swelling clay, which has a self‐healing property for cracks (McDonald, Riha, Duxbury, Steenhuis, & Lauren, 2006). Increased soil organic matter content and NT under CA favours a more stable pore network formation (Li et al., 2019; VandenBygaart, Protz, & Tomlin, 1999). No change in pore characteristics was observed for deeper soil layers. Many authors have reported the effects of tillage only on the upper soil layer (Abdollahi & Munkholm, 2014; Mondal, Poonia, et al., 2020b).

### 4.6 Yield, system productivity and profitability

The rice yield and REY (two crops) were similar or higher under CA in comparison to TA, except for pCA2, which produced significantly lower yield than the other treatments. Better soil aggregation and increased SOC status might have improved nutrient availability (Jat et al., 2019) and microclimatic conditions (Gathala et al., 2013). Pittelkow et al. (2015) have reported a 2.5% yield reduction under CA through a global meta‐analysis. Consistently lower rice yield in pCA2 during the two reported years could be ascribed to rice mealybug infestation (*Brevennia rehi*). A higher proliferation of a grassy weed (*Brachiaria* spp.), which acts as an alternate host of mealybug, was the main reason for mealybug infestation (Mishra et al., 2019). The soil weed seed bank dynamics greatly depends on the cropping system and management practices. The cropping system in pCA2 might have favoured the proliferation of that particular weed and mealybug infestation. Inclusion of the third crop under CA improved the system productivity considerably in comparison to conventional practices and could play a pivotal role in food as well as nutritional security of the region. Higher system productivity also proves the economic viability of CA as a whole (Kumar et al., 2020; You et al., 2017).

## 5 CONCLUSIONS

Our study showed the importance of a CA‐based cropping system in improving soil quality in long run. Minimal soil disturbance, residue retention and inclusion of legumes or crop diversification improved the soil physical parameters (i.e., macroaggregates, MWD and GMD of aggregates, and aggregation ratio). Both SOC concentration and stock improved considerably under a CA‐based cropping system and thus established the role of CA in maintaining better soil health. Organic C was enriched in each aggregate class and no dependency of SOC was observed for aggregate diameter. The maximum contribution of macroaggregates was to SOC stock. The rice yield was similar or higher in the CA‐based cropping system (except for pCA2), whereas SREY was always higher under CA. The effects of rice–wheat cropping systems on soil C are restricted to surface soil layers. Thus, the no‐tillage system and the maintenance of crop residue on the soil surface were effective, even concentrated in a tiny soil layer, in enhancing rice yield and profitability. The traditional rice–wheat systems followed in the Indo‐Gangetic Plain were not suitable for decreasing the soil bulk density (which is very large due to puddling operations) and improving water retention in relation to available soil water. Therefore, some alternative soil management practices should be adopted to improve these soil traits. Therefore, the CA‐based cropping systems can be promoted in the eastern Indo‐Gangetic Plain for sustaining crop productivity with better soil health. The effect of CA on pore‐size distribution was inconclusive and further studies should be undertaken for better understanding of the role of long‐term CA in the soil pore network.

## AUTHOR CONTRIBUTIONS

**Surajit Mondal:** Conceptualization; data curation; investigation; validation; writing‐original draft. **Janki Mishra:** Conceptualization; project administration; writing‐review & editing. **Shis Poonia:** Project administration; resources. **Rakesh Kumar:** Methodology; writing‐original draft. **Rachana Dubey:** Visualization. **Santosh Kumar:** Writing‐review & editing. **Mausam Verma:** Data curation. **Koteswara Rao Karnena:** Data curation; validation. **Akram Ahmed:** Validation. **Sharad Kumar Dwivedi:** Visualization; writing‐review & editing. **Bhagwati Bhatt:** Project administration; writing‐review & editing. **R.K. Malik:** Conceptualization; project administration. **Virender Kumar:** Project administration. **Andrew McDonald:** Project administration.

## CONFLICT OF INTEREST

None.

## Supporting information

**Figure S1**. Location of the experimental site, and monthly four years (2015–16 to 2018–19) average of rainfall, pan evaporation, minimum and maximum temperature during the crop growing seasons. (Source: Agromet Observatory, ICAR‐Research Complex for Eastern Region, Patna, Bihar, India.)

## Data Availability

Data available on request from the authors
