# Supplementary material for: Can yield, soil C and aggregation be improved under long‐term conservation agriculture in the eastern Indo‐Gangetic plain of India?
Source: Eur J Soil Sci. 2021 Feb 18;72(4):1742–61. doi: 10.1111/ejss.13092 (PMC8359171; doi:10.1111/ejss.13092)
Supplement: Supplementary file 1 — Figure S1. Location of the experimental site, and monthly four years (2015–16 to 2018–19) average of rainfall, pan evaporation, minimum and maximum temperature during the crop growing seasons. (Source: Agromet Observatory, ICAR‐Research Complex for Eastern Region, Patna, Bihar, India.) [file EJSS-72-1742-s001.docx]

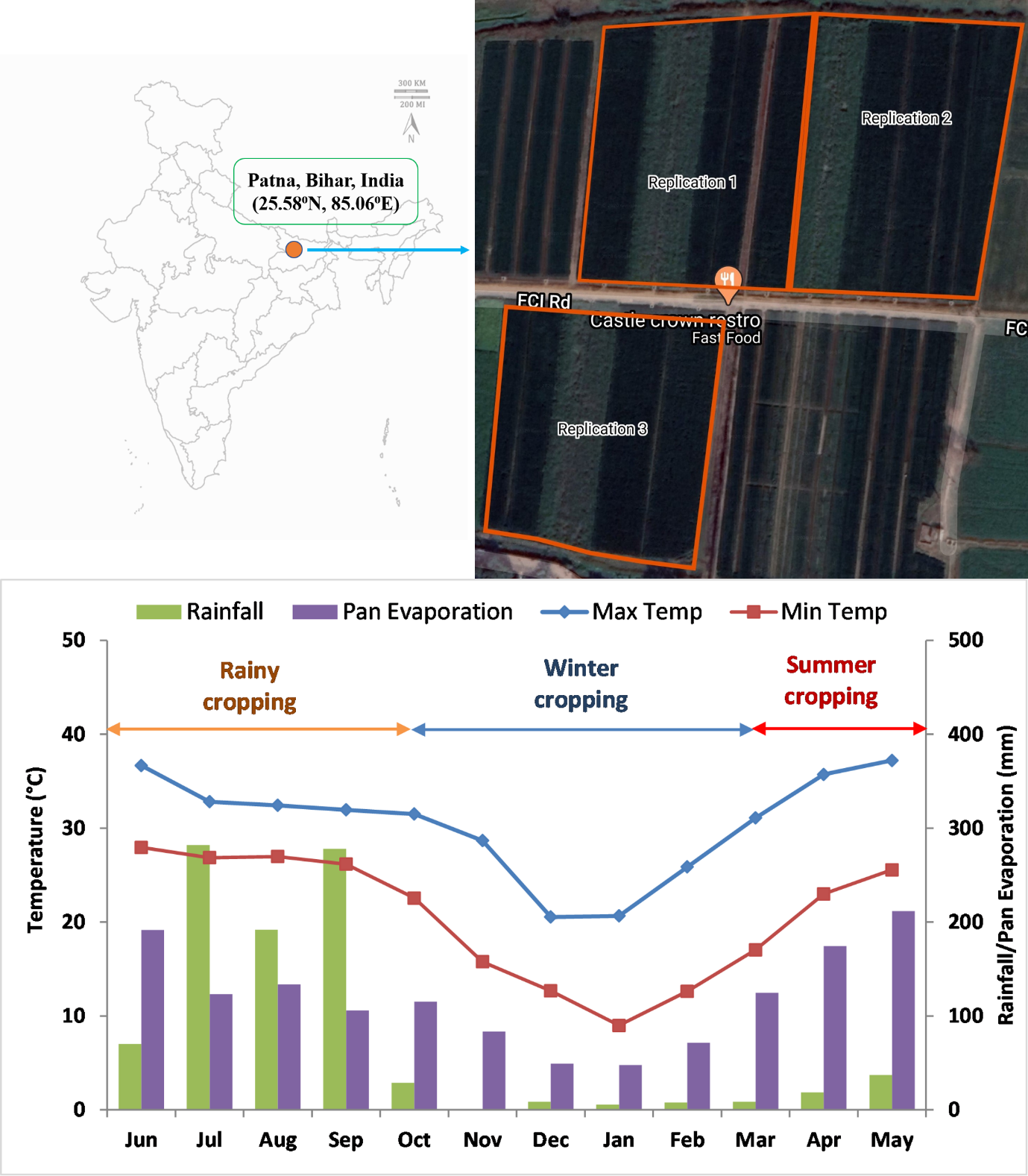


**Supplementary Figure 1:** Location of the experimental site; and monthly four years (2015-16 to 2018-19) average of rainfall, pan evaporation, minimum- and maximum temperature during the crop growing seasons. [Source: Agromet Observatory, ICAR-Research Complex for Eastern Region, Patna, Bihar, India].
